# How will early onset scoliosis surgery affect my child’s future as a young adult? A follow-up study using patient-reported outcome measures

**DOI:** 10.1007/s43390-024-00910-2

**Published:** 2024-06-10

**Authors:** Dmitri A. Falkner, Kyle J. Miller, John B. Emans, George H. Thompson, John T. Smith, Jack M. Flynn, Jeffrey R. Sawyer

**Affiliations:** 1 Campbell Clinic, Department of Orthopaedics, University of Tennessee, Campbell Clinic, 1211 Union Avenue, Suite 510, Memphis, TN 38104 USA; 2 Gillette Children’s Pediatric Orthopedics, Saint Paul, MN USA; 3 Boston Children’s Hospital, Boston, MA USA; 4 Pediatric Spine Study Group, University Health-Rainbow Babies and Children’s Hospital, Cleveland, OH USA; 5 University of Utah, Salt Lake City, UT USA; 6 Children’s Hospital of Philadelphia, Philadelphia, PA USA

**Keywords:** Early onset scoliosis, Idiopathic scoliosis, Spine surgery, Health-related quality of life

## Abstract

**Purpose:**

Using patient-reported outcome measures (PROMs), this study was undertaken to determine how well patients with early onset scoliosis (EOS) fare in adulthood.

**Methods:**

Among eight healthcare centers, 272 patients (≥ 18 years) surgically managed for EOS (≥ 5 years) completed the Scoliosis Research Society (SRS)-22r, Functional Assessment of Chronic Illness Therapy-10 (FACIT-Dyspnea-10), and Short Form (SF)-12. Functional and demographic data were collected.

**Results:**

The response rate was 40% (108/272). EOS etiologies were congenital (45%), neuromuscular (20%), idiopathic (20%) syndromic (11%), and unknown (4%). All patients scored within normal limits on the FACIT-Dyspnea-10 pulmonary (no breathing aids, 78%; no oxygen, 92%). SF-12 physical health scores and most SRS-22r domains were significantly decreased (*p* < 0.05 and *p* < 0.001, respectively) compared with normative values. SF-12 and SRS-22r mental health scores (MHS) were lower than normative values (*p* < 0.05 and *p* < 0.02, respectively). Physical health PROMs varied between etiologies. Treatment varied by etiology. Patients with congenital EOS were half as likely to undergo definitive fusion. There was no difference between EOS etiologies in SF-12 MHS, with t scores being slightly lower than normative peers.

**Conclusion:**

Good long-term physical and social function and patient-reported quality of life were noted in surgically managed patients. Patients with idiopathic EOS physically outperformed those with other etiologies in objective and PROM categories but had similar MHS PROMs. Compared to normative values, EOS patients demonstrated decreased long-term physical capacity, slightly lower MHS, and preserved cardiopulmonary function.

**Level of evidence:**

Level IV Case Series.

## Introduction

Early onset scoliosis (EOS) is a complex condition affecting children 9 years of age and younger and can be classified based on etiology (idiopathic, congenital, syndromic, or neuromuscular) [1, 2]. EOS often is severe and, if left untreated, may be fatal. Furthermore, severe spinal deformity leads to pulmonary hypertension and cor pulmonale; therefore, early treatment of progressive curves is vital to preserving cardiopulmonary function [3]. Initial treatment of EOS can consist of spine-based as well as rib-based growing constructs [4, 5]. Although numerous radiographic studies exist comparing these techniques, little data exist on the long-term quality of life and social functioning of patients with EOS as they reach adulthood [5, 6].

By combining patient demographics with disease-specific and generic health-related quality of life (HRQoL) questionnaires, this study sought to determine the long-term medical and social outcomes and HRQoL in adult patients with EOS [7]. The Scoliosis Research Society 22-Item Revised (SRS-22r) questionnaire, which has been validated for adult patients [8], the Short Form-12 (SF-12), and the Functional Assessment of Chronic Illness Therapy-Dyspnea item-10 (FACIT-Dyspnea-10), which has been validated in chronic obstructive pulmonary disease (COPD), systemic sclerosis, and scleroderma [9–13], were used to collect spine-specific, generic physical and mental, and pulmonary function HRQoL, respectively, in adult patients with EOS.

The purpose of this study was to determine the quality of life in adult patients who were treated surgically in childhood for EOS. This important information will help clinicians better advise patients with EOS and their families on what to expect as they enter adulthood, and it will assist in developing a methodology to study this valuable patient cohort as they age.

## Materials and methods

### Patient selection

**Fig. 1:**
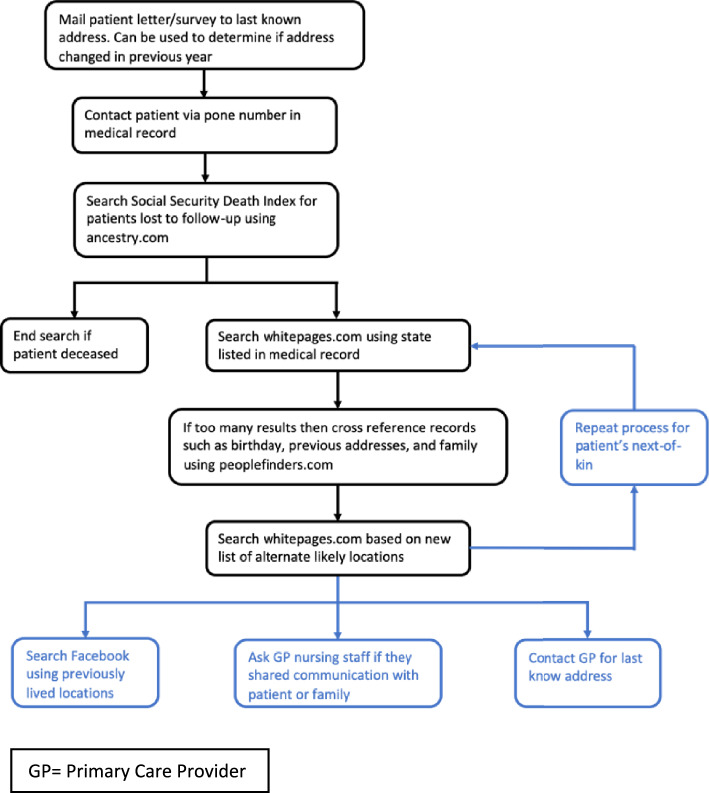
Search algorithm: overall response rate 40%

After obtaining approval from each center’s institutional review board, retrospective chart review from eight healthcare institutions was performed of adult patients (18 years and older) who were treated for EOS and were at least 5 years from their most recent surgery. Personal details including name, date-of-birth, home address, email address, phone number, and general practitioners’ office phone were recorded. Patients or caregivers were contacted using a comprehensive search algorithm that included mailing address and phone number on file, Social Security Death Index, online phone and people search directories, social media, and general practitioners’ offices (Fig. 1).

### Data collection and outcome measures

Patients and/or their caregivers, when patients were unable, were instructed to fill out three separate patient-reported outcome measures (PROMs): the SRS-22r, the SF-12, and the FACIT-Dyspnea-10. Data collection occurred between 11/2019 and 3/2021. Patient demographics were recorded, including age, gender, and etiology of EOS. Additional functional data were collected, including education level, employment status, marital/parental status, use of mobility and/or breathing aids, and oxygen requirements.

### Statistical analysis

SRS-22r scores were compared between EOS patients and normative respondents for the corresponding PROMs using a Student *t*-test. FACIT-Dyspnea-10 and SF-12 scores were analyzed using a *T*-score metric with a normative mean of 50. Statistical significance was defined at the *α* = 0.05 level.

## Results

**Fig. 2:**
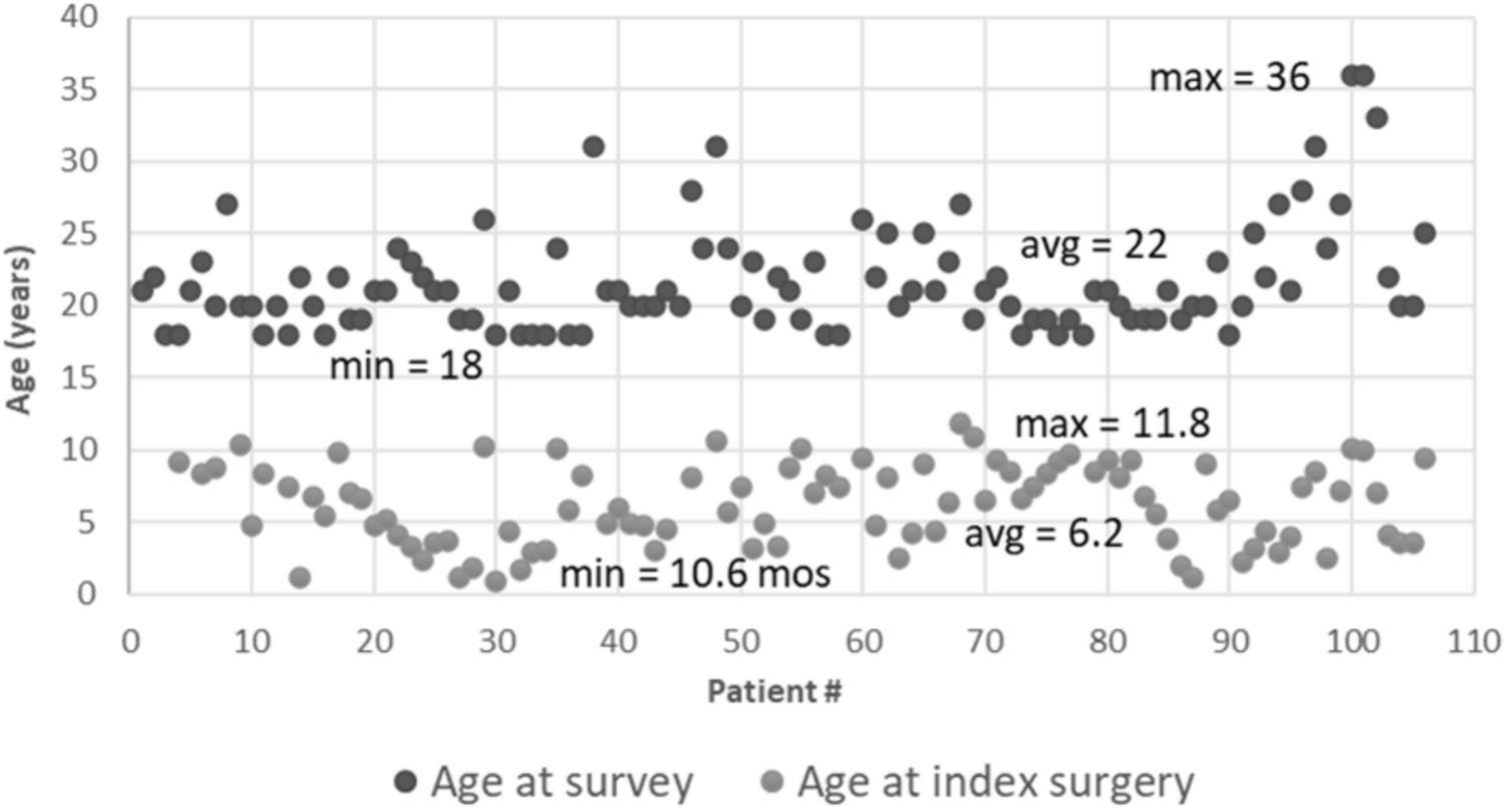
Patient specific age at index procedure and follow-up

**Fig. 3:**
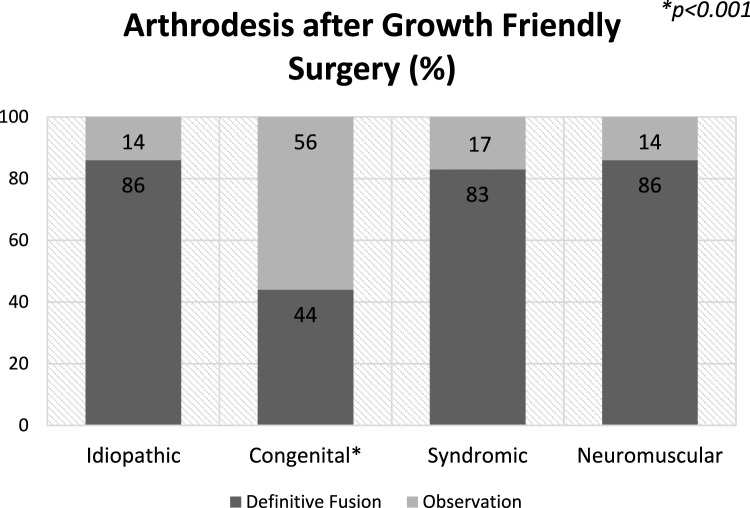
Treatment by etiology

**Fig. 4:**
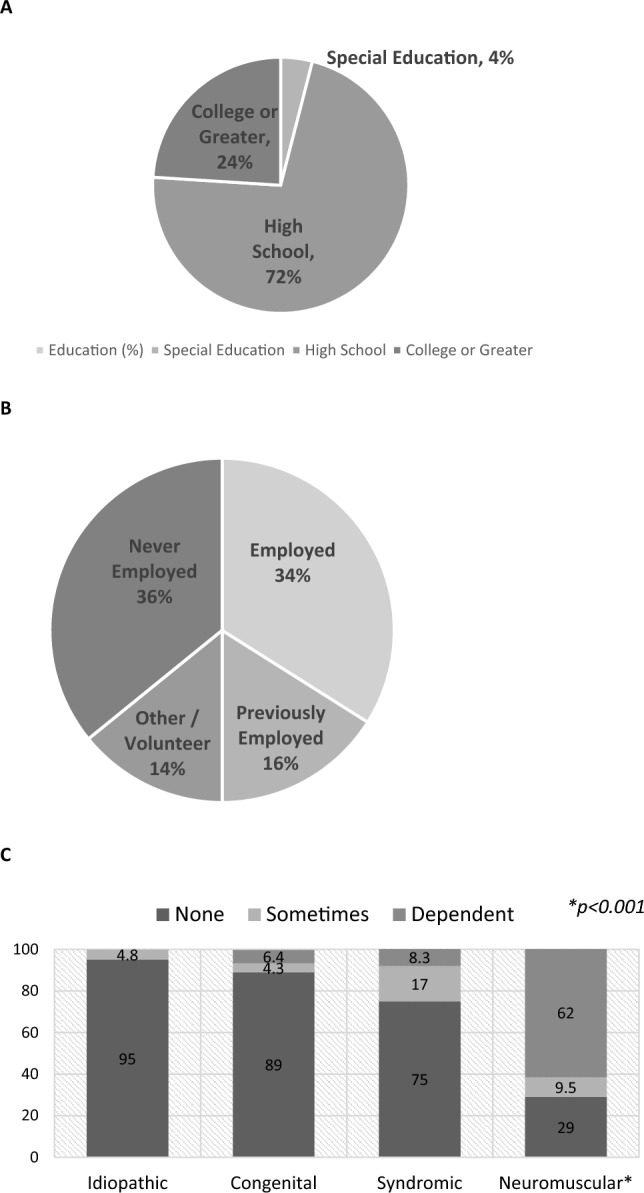
Social function. **A** Highest educational level, **B** employment history, **C** mobility: wheelchair dependence

Two hundred and seventy-two patients were identified and 108 (40%) agreed to participate; 3 (0.011%) were deceased. Seventy-seven percent of the questionnaires were completed by the patient, 20% by the caregivers, and 2% by both. The method of completion was based on patient distance/convenience and included in-person, telephone, or email. The decision regarding whether the patient or the guardian filled out the outcome measures was determined by the patients’ co-morbidities, if present, and the investigator. Patient ages ranged from 18–36 (mean 22) years, and 58 (54%) were female (age at the time of surgery and survey and etiologies are noted in Table 1 and Fig. 2). The treatment method and decision to perform arthrodesis were at the discretion of the senior surgeon. Arthrodesis was performed in 67 patients (62%), with a mean of 6.7 years between the index procedure and arthrodesis. Treatment varied by EOS etiology (*p* < 0.001). Patients with congenital EOS were half as likely to undergo definitive fusion as other etiologies (Fig. 3). Overall, patients were found to be quite functional socially (Fig. 4); 100 patients (96%) completed high school or higher education, 25 (23%) completed college including one completing medical school, 37 (34%) were presently employed, and 69 (64%) had at some point either been employed or worked in a volunteer capacity. Seventy six patients (69%) mobilized without any assistive devices, 17 (16%) ambulated with an assistive device and 15 (14%) required a wheelchair. Three patients were married and three patients had children.

**Table 1:** Demographics (*n* = 108) <

Characteristic	
Gender: Female:Male	58 (54%): 47(46%)^a^
Age at index procedure (years)	6.2 (range 0.9–11.8 years)
Age at follow-up (years)	22 (range 18–36 years)
Etiology	
Congenital	49/45%
Idiopathic	20/20%
Neuromuscular	21/20%
Syndromic	12/11%
Other	6/5%

^a^3 patients did not report

**Fig. 5:**
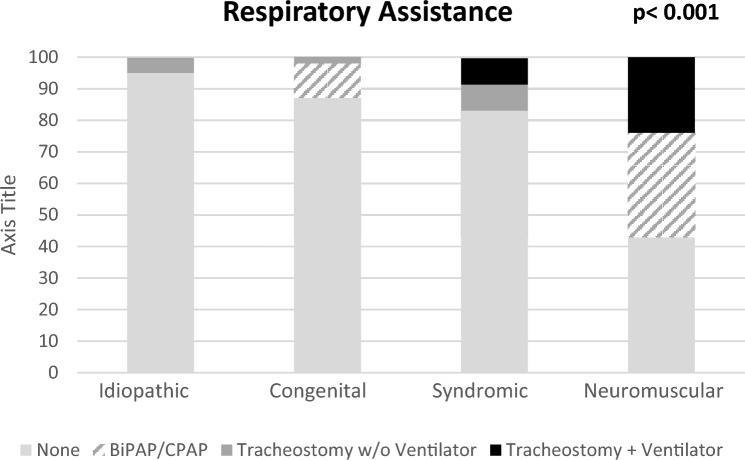
Respiratory assistance

Although 33 patients (31%) noted some limitations due to breathing issues, overall pulmonary function was found to be quite good (Fig. 5). All patients scored within normal limits on the FACIT-Dyspnea-10 questionnaire: 86 (80%) used no breathing aids; 12 (11%) used occasional bilevel or continuous positive airway pressure (BiPAP/CPAP); 3 (3%) required a tracheostomy alone; 6 (6%) required tracheostomy and a ventilator; 99 (92%) had no oxygen requirement; 6 (6%) required occasional oxygen; and 2 (2%) required continuous oxygen.

**Fig. 6:**
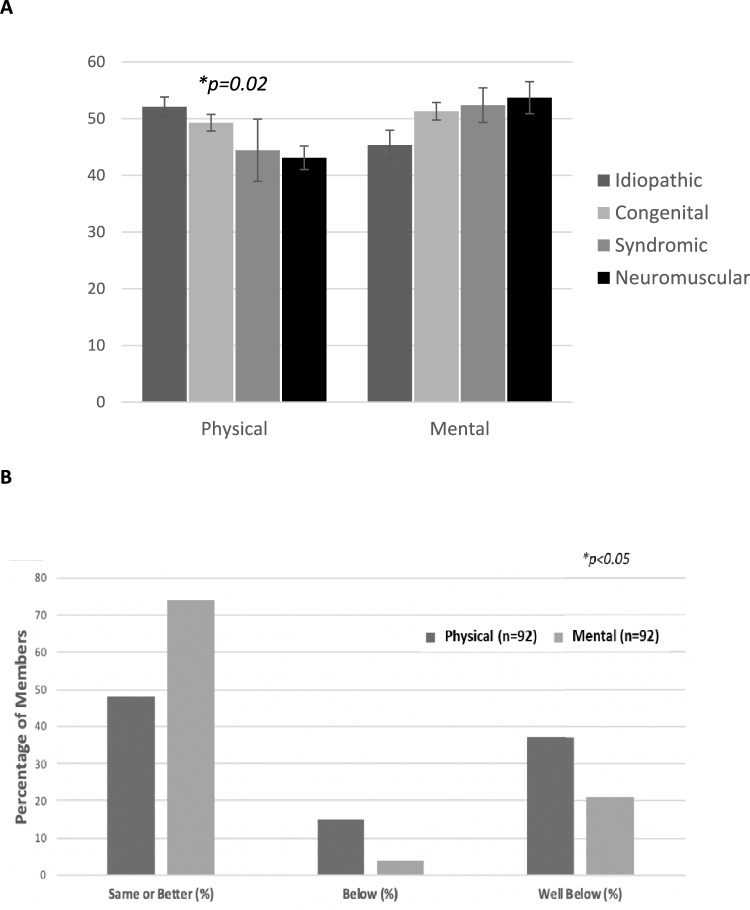
**A** Short Form-12 (SF-12) outcomes by domain and etiology, **B** SF-12 component compared to normative data

**Fig. 7:**
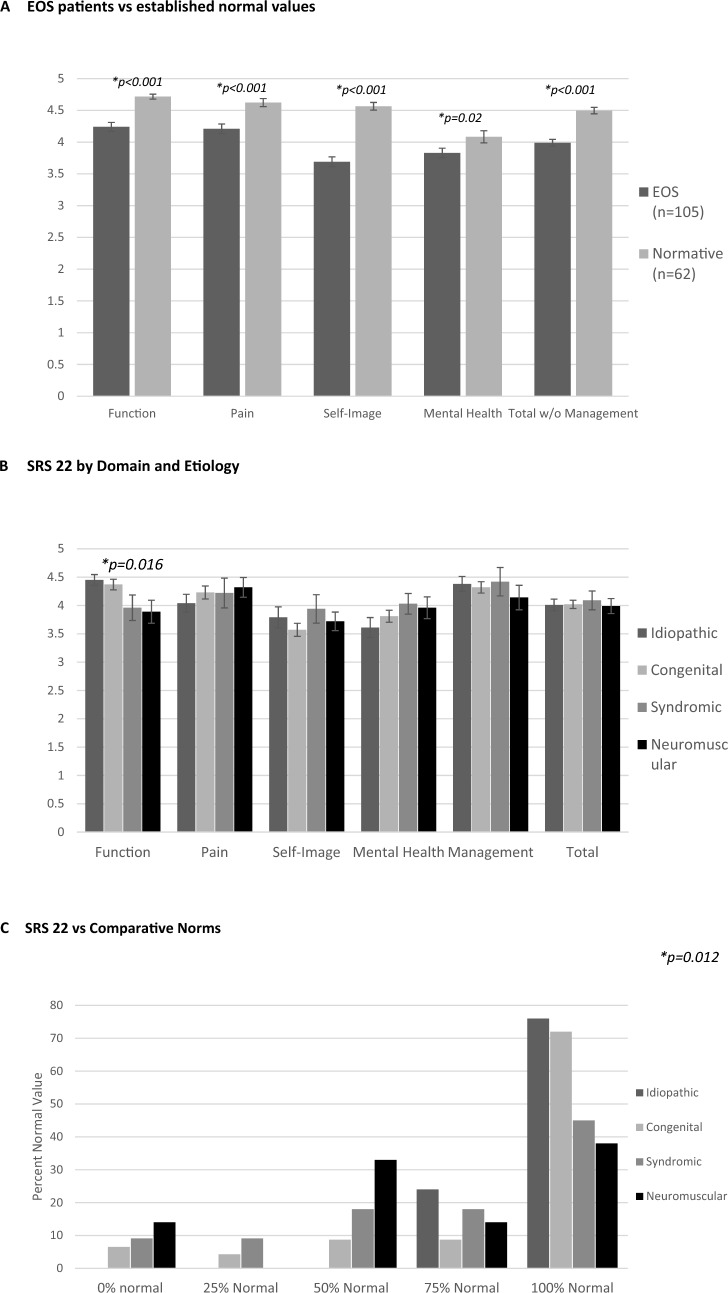
Scoliosis Research Society 22 (SRS-22r) outcomes. **a** Early onset scoliosis patients compared with established norms, **b** SRS-22 domain and etiology, **c** SRS-22 comparative norms

Patients with EOS and their caregivers acknowledged the role that physical ability and pain play in day-to-day function. The SF-12 component summary physical health scores as well as most SRS-22r domains (including function, pain, and self-image) were significantly decreased (*p* < 0.05 and *p* < 0.001, respectively) for patients with EOS when compared to normative values (Figs. 6 , 7). Despite these potential physical disabilities, patients with EOS appeared to be mentally and emotionally well-adjusted. Both the SF-12 component summary mental health scores (*p* < 0.05) and the SRS-22r mental health domain (*p* = 0.02) for patients with EOS were only slightly decreased when compared to normative values. No statistically significant correlation was noted between the number of surgical procedures and HRQoL questionnaire outcomes.

EOS etiology was significantly associated with a number of physical health indicators as well as physical health PROMs. Patients with idiopathic EOS were of a greater height (*p* < 0.05) and weight (*p* < 0.001) and were more likely to self-respond (*p* < 0.05) and be employed (*p* < 0.03) when compared to patients with other EOS etiologies. They also reported better SF-12 component summary physical health scores (*p* = 0.02) than other etiologies; patients with neuromuscular EOS reported the lowest scores. A majority of patients with neuromuscular EOS (*p* = 0.012) rated their current level of function as half of normal population values or less based on SRS-22r score (Fig. 7C). Ambulatory independence was significantly (*p* < 0.001) associated with EOS etiology, with 19/20 (95%) of patients with idiopathic, 43/49 (89%) with congenital, 8/12 (75%) with syndromic, and 6/21 (29%) with neuromuscular EOS requiring no wheelchair (Fig. 4C). Additionally, the use of breathing aids was significantly (*p* < 0.001) different between etiologies: 19 of 20 patients (95%) with idiopathic, 42 of 49 (87%) with congenital, 10 of 12 (83%) with syndromic, and 9 of 21(43%) with neuromuscular EOS required no breathing aids (Fig. 5). Treatment also varied by EOS etiology (*p* < 0.001); patients with congenital EOS were half as likely to undergo definitive fusion (Fig. 3). Despite co-morbidities, there were no significant differences between EOS etiologies for SF-12 component summary mental health scores (Fig. 6).

## Discussion

This retrospective study used a large multi-center database of surgically managed EOS and evaluated long-term HRQoL in patients using three different PROMs: the SRS-22r, the SF-12, and the FACIT-Dyspnea. Although we recognize that other PROMs could have been used, these were chosen to determine overall EOS patient quality of life while attempting to prevent survey/respondent fatigue [14, 15].

In the current study, patients with EOS demonstrated decreased physical function for both the SF-12 and SRS-22r function domains when compared with normative peers. When stratified by etiology, patients with idiopathic and congenital EOS scored higher than those with syndromic or neuromuscular EOS. These findings are similar to those of Matsumoto et al.[16] and are not surprising because many patients with neuromuscular and/or genetic conditions have other co-morbidities that affect ultimate quality of life measures in adulthood. Yildiz et al. also reported similar long-term SRS-22 scores in a small cohort of patients (*n* = 15, mean age 18.7 years) [17]. It is encouraging, however, that given the large number of surgical procedures and complications associated with the treatment of EOS, these patients are functioning well socially in adulthood. Although significant limitations remain in the physical domains of their SF-12 measures, approximately 70% have mental health scores the same or better than normative peers.

Patients with EOS had slightly lower mental health scores than normative data for both the SF-12 and the SRS-22r, but although statistically significant these differences were comparatively mild clinically. A majority of patients with EOS scored the same or better than normative data on the SF-12. This is in contrast to prior studies demonstrating higher levels of adverse psychologic outcomes with EOS, particularly with repetitive surgery [18, 19]. Yildiz et al. reported psychologic abnormalities in two-thirds of 15 patients from a center in Turkey, but these were mild, and the authors did not report social functioning. Cultural differences in surgical expectations or outcomes and the translation of the SRS-22 could account for the difference [17, 20]. The findings in the current study are more in agreement with those of Vitale et al. who demonstrated similar psychosocial scores between patients with EOS and thoracic insufficiency and normative data [21]. It is possible that improved mental health scores in the current study are a result of the long-term follow-up, which allowed patients more time to recover psychologically from the trauma of surgery, or perhaps patients were able to develop positive coping strategies [22]. A follow-up survey of this cohort is planned to determine how these results might change over time.

Some signs of pulmonary dysfunction were noted; 31% noted some limitations in activities secondary to breathing issues. Although dyspnea severity and functional limitations were within normal limits on the FACIT-Dyspnea-10 score, breathing aids were needed in a majority of patients with neuromuscular EOS but only rarely in patients with idiopathic EOS, which agrees with the findings by Matsumoto et al. who reported better outcomes with idiopathic and congenital EOS than neuromuscular or syndromic EOS [16].

Demographics collected in this study provide an interesting insight into how patients with EOS compare socially to normative peers. The mean patient age in this study was 22 years, with only three patients being married and three having children. These findings are not surprising given that the 2021 United States Census median age at marriage was 29 years and the mean age at first birth in 2015 was 23 for females and 25.5 for males [23, 24]. Employment data are harder to evaluate because most of the responses were collected during a time of historic unemployment secondary to the COVID-19 pandemic. Compared with 46.7% of United States citizens (ages 16–24 years), 34% of patients with EOS were employed in 2020 [25]. Further long-term follow-up is needed to determine how patients with EOS ultimately compare with societal norms.

### Strengths and limitations

The current study represents the largest reported cohort of patients with EOS with follow-up into early adulthood. Other strengths include the relatively heterogeneous population, the wide array of etiologies, and the multi-center design that make these results generalizable. The use of multiple PROM questionnaires is another advantage; most long-term follow-up studies report only radiographic parameters or unplanned return to the operating room. This study also is the first to focus on how young adult patients with EOS are truly doing in terms of health and social functioning. This information is critical for patients, families, and health care providers to be able to understand and plan for adult life when surgical treatment for EOS begins, typically at a young age.

Achieving good response rates for surveys conducted after a long period with no or minimal patient contact remains a challenge, including difficulty locating patients and their unwillingness to participate [26–29]. The literature indicates that long intervals of no contact does not preclude follow-up, and most patients contacted are willing to participate [30, 31]. Older age also has been associated with improved follow-up in long-term surveys [32]. A target response rate of 40% was chosen for this study based on findings that 60% attrition due to randomness does not impart any important statistical bias [33]. The search algorithm for this project was based on that developed by Louie et al. that included using web-based people search platforms (Fig. 1) [30]. We added the component of contacting the patient’s general practitioner to obtain information on patients’ locations [34]. This resulted in a patient follow-up of 40%. Given our average patient response age of 22, higher attrition could reasonably be expected in this group. Another potential confounder is the exclusion of deceased patients, which would have represented a more debilitated population. They were excluded because the purpose of this study was to evaluate HRQoL in long-term survivors. In addition, our mortality rate of around 1% would represent a very small proportion of the overall cohort.

An additional concern is in selecting the appropriate PROMs to obtain relevant information for HRQoL. These must be balanced in regard to amount and length of surveys to minimize respondent fatigue [14, 15]. When possible, well-validated and disease-specific tools should be used. At the time of development of this study, no EOS-specific PROMs had been validated in older children or adults; therefore, other validated adult outcome studies were chosen. To capture more of the patient’s experience [35], we chose the SRS-22r questionnaire because of its widely accepted use in assessing HRQoL for a variety of spine conditions, including congenital scoliosis [36]. Its primary use has been adolescent idiopathic scoliosis [37], but it also has been used for long-term spine PROM in adults [8, 38]. The SRS-22r has been shown to be more specific for spine-related conditions than the SF-12 [39]; however, we included the SF-12 to capture components missed by the SRS-22r. The SF-12 has been used for a number of spine conditions but is not as well-documented in the EOS literature. It has been used with other spine-specific HRQoL questionnaires to evaluate long-term PROMs in spine patients [38, 40]. The SF-12 was also included because of its brief yet comprehensive assessment of the physical and mental HRQoL. The FACIT-Dyspnea-10 was used for assessment of pulmonary function. It was developed and validated in chronic obstructive pulmonary disease (COPD) [9–11]. It has not been validated in spine patients but has been in systemic sclerosis, which presents as restrictive lung disease resembling that which occurs with EOS [12, 13]. Interestingly, low FACIT-Dyspnea-10 scores (and thus good patient-reported pulmonary HRQoL) have not been shown to correlate well with pulmonary function testing [10, 41]. The early onset scoliosis questionnaire (EOSQ) was not used because it focuses on parental and financial burden in childhood EOS [42]. More recently, the Early Onset Scoliosis Self-Report Questionnaire (EOS-SELF) was developed to target mature EOS populations to determine long-term HRQoL [43]. Further evaluation and validation of this PROM may help yield even more standardized and disease-specific data on this unique population.

Considerable heterogeneity exists in the EOS population in terms of etiology, age, co-morbidities, and treatment methods used and it is a rare condition. As our patient base grows and reaches adulthood, we will be able to further stratify and analyze outcomes based on other parameters such as curve magnitude/correction, treatment method, number of surgical procedures, and their correlation to long-term outcomes. Even more patients will be necessary for meaningful statistical sub-group analysis. Despite these limitations, our findings will provide answers to fundamental questions families have about social functioning, independence, and quality of life in adulthood after EOS treatment.

Current treatments for EOS have allowed patients to overcome what was once a devastating and often terminal condition early in life, and now patients are frequently living into adulthood. Numerous studies have assessed long-term radiographic outcomes in patients with EOS, but this is the first study to assess social and global health functioning as determined by disease-specific PROMs. Overall, EOS patients in early adulthood have good long-term physical and mental self-reported function and preserved self-reported pulmonary function, with the majority requiring no mobilization or breathing aids. Long-term follow-up will be useful to see how they continue to develop and function in society. This work highlights the importance of using PROMs in assessing the long-term function to follow this cohort into later adulthood.

## Data Availability

The data is housed and available by request from the Pediatric Spine Study Group.
